# The Intrinsic Disordered N-Terminus of Nucleocapsid Protein of SARS-CoV-2 Is Critical in DNA Aptamer Binding

**DOI:** 10.3390/ijms27146386

**Published:** 2026-07-18

**Authors:** Hongye Lu, Jiawen Ma, Xiaomin Ma, Yuanpeng Wu, Xuan Sun, Changxing Ma, Xiaoxian Li, Zhiyong Xu, Pengxi Lu, Zhaofeng Luo, Liyun Zhang, Lixin Zhang, Shenlin Wang

**Affiliations:** 1 State Key Laboratory of Bioreactor Engineering, East China University of Science and Technology, Shanghai 200237, Chinamarco.xu2001@outlook.com (Z.X.);; 2 Instrumental Analysis and Research Center, Sun Yan-sen University, Guangzhou 510006, China; 3 State Key Laboratory of Microbial Metabolism, Joint International Research Laboratory of Metabolic and Developmental Sciences, School of Life Sciences and Biotechnology, Shanghai Jiao Tong University, Shanghai 200240, China; 4 School of Life Sciences, University of Science and Technology of China, Hefei 230027, China; 5 State Key Laboratory of Medicinal Chemical Biology, Nankai University, Tianjin 300071, China; 6 Beijing NMR Center and College of Chemistry and Molecular Engineering, Peking University, Beijing 100871, China

**Keywords:** nucleocapsid protein (N protein), DNA aptamer, A48, protein-nucleic acid interaction, intrinsically disordered region (IDR), SARS-CoV-2, NMR

## Abstract

SARS-CoV-2 nucleocapsid protein (N protein) binds nucleic acids and packages viral RNA. DNA aptamers that specifically bind the N protein have been used in antigen-based COVID-19 detection and have potential clinical applications for preventing SARS-CoV-2 infection. However, the complex structures of the N protein with DNA aptamers and the mechanisms by which aptamers recognize the N protein remain unclear. Here, we report the NMR-derived complex structure of the N-terminal domain of the N protein (N-NTD) with a 58 nt DNA aptamer, A48. The complex structure reveals a distinct topology with a large contact area between A48 and N-NTD. The N-terminal intrinsically disordered region (IDR) of N-NTD forms close contact with A48, primarily stabilized by hydrophilic interactions. Deletion of the N-terminal IDR or substitution of positively charged arginine residues with negatively charged glutamate residues in the IDR region substantially reduced the binding affinity for A48. Because most previously determined N protein structures were obtained using constructs lacking the N-terminal IDR, this study reveals a topology of the N protein-nucleic acid complex and highlights the importance of the N-terminal IDR in nucleic acid binding.

## 1. Introduction

The nucleocapsid protein (N protein) of SARS-CoV-2 is one of the most abundant structural proteins in the virus and functions in packaging the viral genome inside the virion [1,2,3,4]. It also interferes with signaling pathway proteins, facilitates immune evasion, and hyperactivates immune-related cytokine expression, leading to severe immune responses [5,6,7,8,9,10]. Lead compounds targeting the N protein have been developed to block its binding to interaction partners. In addition, various monoclonal antibodies and DNA aptamers have been discovered for accurate and efficient COVID-19 diagnosis [11,12,13,14,15]. Therefore, characterizing the structure, interactions, and functions of the N protein may provide insights into the viral life cycle and offer clues for drug development against SARS-CoV-2 infection. 

Structural characterization of the complex between the N protein and its DNA aptamer could provide valuable insight into the recognition mechanism and facilitate the rational design of new aptamers or related drugs [16,17]. To date, a series of DNA aptamers targeting the SARS-CoV-2 N protein have been reported. These aptamers bind the N protein through electrostatic forces, hydrogen bonding, van der Waals interactions, and characteristic dissociation kinetics [14,17,18,19]. Among the published aptamers, A48 and A58 exhibit very high affinity for the N protein, with equilibrium dissociation constant (*Kd*) in the subnanomolar range [16].

In this study, we used NMR spectroscopy to identify the key binding sites of the N protein with the 58 nt DNA aptamer A48. The N-terminal domain of the N protein, but not the C-terminal domain, bound A48 with nanomolar affinity, similar to that of the full-length N protein [17]. NMR titration experiments demonstrated that N-NTD binds A48 primarily through hydrophilic interactions. Both the highly positively charged basic finger motif and the intrinsically disordered N-terminus of N-NTD are critical for DNA aptamer binding. Deletion of the flexible N-terminal segment or single-point mutants of arginine (Arg) residues in this region altered the binding mode and substantially reduced the binding affinity for A48. In contrast, arginine-to-glutamate (Arg-to-Glu) mutants in the basic finger motif had much smaller effects on A48 binding. Structural analysis of the N-NTD-A48 complex revealed that the flexible N-terminus folds into a β-strand structure upon aptamer binding, suggesting a cooperative binding and folding recognition mechanism. Unlike most structural studies in which the flexible N-terminus of the N protein was excluded, this work highlights the importance of the flexible N-terminus in nucleic acid binding and provides clues for the rational design of DNA aptamers targeting the N protein.

## 2. Results

### 2.1. A48 Aptamer Binds to the N-Terminal Domain of Nucleocapsid Protein 

**Figure 1:**
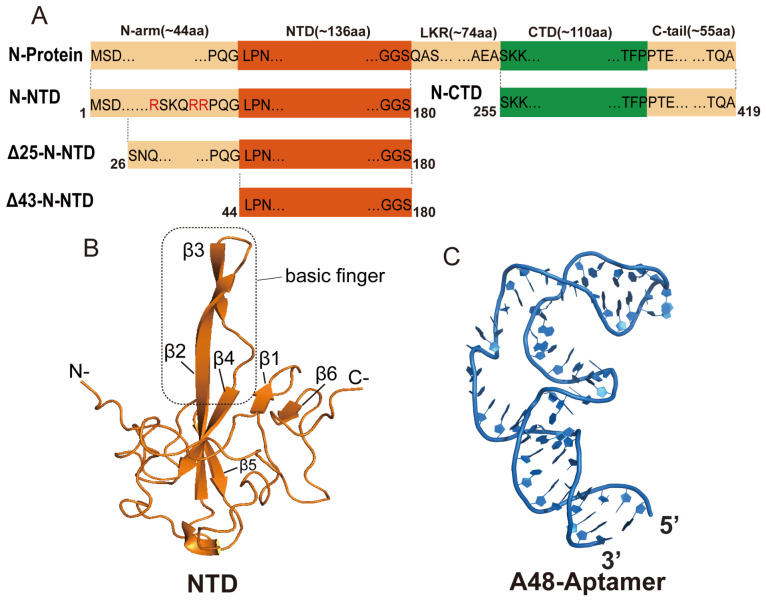
Domain organization and structural characterization of nucleocapsid protein (N protein) and A48 aptamer. (**A**) Schematic diagram of the N protein domains. The truncation constructs used in this study are indicated. (**B**) Structural model of the N-terminal domain (N-NTD) in solution of this work, which was built from crystal structure of N-NTD (PDB code: 7CDZ). (**C**) Three-dimensional structure of the A48 aptamer determined by molecular dynamics simulations guided by NMR-derived restraints. (see main text for details).

We characterized the key region of the nucleocapsid protein (N protein) involved in binding to A48, a previously identified single-stranded DNA aptamer. The N protein contains 419 amino acid residues and comprises two structural domains: the N-terminal domain (N-NTD, residues 1–180) and the C-terminal domain (N-CTD, residues 255–419) (Figure 1A). Both domains contain regions predicted to bind nucleic acids. High-resolution structures of both folded domains have been solved independently, and the N-NTD has been shown to bind RNA via a highly positively charged basic finger motif (Figure 1B), corresponding to the β2–β4 region [20]. Additionally, the protein features three intrinsically disordered regions that serve as connectors between the two domains and at their flanks [21,22,23]. In addition, we predicted the three-dimensional structure of A48 using molecular dynamics simulations, as shown in Figure 1C. The aptamer adopts an overall L-shaped topology.

Surface plasmon resonance (SPR) experiments were performed to identify the domain responsible for A48 binding. In this study, N-NTD bound A48 with a *Kd* value of 0.36 nM, which is comparable to the binding affinity of the full-length N protein (*Kd* = 0.49 ± 0.05 nM) [16]. In contrast, no binding was detected between N-CTD and A48 (Supplementary Figure S1). These results demonstrate that N-NTD, not N-CTD, is the A48-binding domain.

### 2.2. NMR Spectroscopy Identifies the N-Terminal Intrinsically Disordered Region and Basic Finger Region as A48 Binding Epitopes

To identify the regions involved in the interaction between N-NTD and A48, we recorded ^1^H-^15^N HSQC spectra of free N-NTD and the N-NTD-A48 complex. The complex was formed using ^15^N-labeled N-NTD and unlabeled A48. In the free state, 98 of 180 residues were detected and assigned (Supplementary Figure S2). The unassigned residues are primarily located in the N-terminal segment (M1–T49) and the basic finger region (T91–K102), consistent with these regions being intrinsically disordered or adopting multiple conformations in the absence of A48. This observation agrees with the prediction that the N-terminal region functions as an IDR. Chemical shift index (CSI) analysis of the assigned residues revealed a predominance of β-strand structures, consistent with previous crystal structures of the N protein and the solution structure of N-NTD bound to RNA [2].

**Figure 2:**
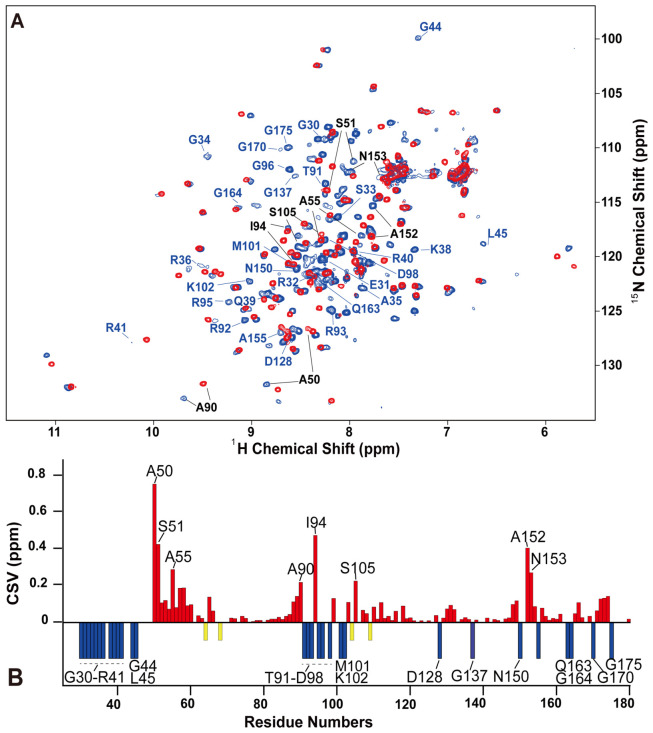
HSQC spectral comparison and chemical shift variation (CSV) analysis of N-NTD and N-NTD-A48. (**A**) Comparison of ^1^H-^15^N HSQC spectra of N-NTD before (red) and after (blue) the addition of A48. Blue labels indicate signals that were absent in the free N-NTD spectrum but emerged upon A48 addition. Black labels denote signals that exhibited substantial chemical shift changes (CSV > 0.3 ppm) after A48 addition. (**B**) CSV analysis of N-NTD residues before and after A48 binding. Red bars indicate residues that experienced chemical shift changes upon A48 binding. Blue bars indicate residues that were newly detected in the N-NTD-A48 complex. Yellow bars indicate residues that disappeared after A48 binding.

Upon A48 binding, substantial spectral changes were observed (Figure 2A). Approximately 30 additional residues became detectable in the A48-bound state (Figure 2A and Supplementary Figure S3). Residues G30–S51 (N-terminal IDR) and T91–K102 (basic finger region) showed either newly observed signals or substantial chemical shift changes (chemical shift variation > 0.3 ppm). In addition, several residues in the loops connecting β-strands—including D128, G137, N150–A155, G163, G164, G170, and G175—became discernible in the presence of A48. Collectively, these data demonstrate specific recognition of A48 by the G30–S51 region of the N-terminal IDR and the T91–K102 region of the basic finger domain. Furthermore, A48 binding induces a more compact and rigid conformation of N-NTD, indicating that A48 influences the structural dynamics of the protein.

Previous studies have indicated the importance of the basic finger region of N-NTD for RNA/DNA binding. However, those studies used a truncated form of N-NTD lacking the N-terminal IDR (residues 1–43) [2,24,25,26]. Therefore, the specific contribution of the N-terminal IDR to nucleic acid binding remained unclear.

To further validate the role of the N-terminal IDR in A48 binding, we generated two N-terminally truncated N-NTD variants: Δ25N-NTD (residues 26–180) and Δ43N-NTD (residues 44–180). Both constructs exhibited significant spectral changes upon A48 addition, indicating interaction with A48. Comparative analysis showed that the spectra of the Δ25N-NTD-A48 complex closely resembled those of the full-length N-NTD-A48 complex, confirming that residues 1–25 do not participate in or influence the binding between N-NTD and A48 (Supplementary Figure S4A). In contrast, the Δ43N-NTD-A48 complex displayed notable differences compared with the full-length complex (Supplementary Figure S4B). This observation highlights the critical role of residues S26–Q43 within the N-terminal IDR in mediating A48 binding, a conclusion supported by backbone chemical shift variation (CSV) analysis.

The Δ25N-NTD-A48 and Δ43N-NTD-A48 complexes exhibited *Kd* of 3.09 nM and 5.65 nM, respectively. Notably, Δ43N-NTD displayed an approximately 1.8-fold reduction in binding affinity compared with the Δ25N-NTD, suggesting that this N-terminal IDR segment contributes to optimal aptamer recognition. These data underscore the functional importance of the N-terminal IDR in maintaining efficient interaction with A48.

### 2.3. Hydrophilic Interactions Determine the Binding Between N-NTD and A48 and the Structure of the N-NTD-A48 Complex

We further identified critical residues within the N-NTD-A48 complex that serve as hotspots and influence the structural arrangement of the complex. In the spectral region corresponding to guanidine NH groups (indicated by a ^15^N chemical shift of 80 ppm in the HSQC spectrum), the interaction with A48 triggered the emergence of seven distinct new signals. This observation suggested the potential involvement of multiple Arg residues in intermolecular salt bridges or hydrogen bonds, contributing to direct interaction with A48.

**Figure 3:**
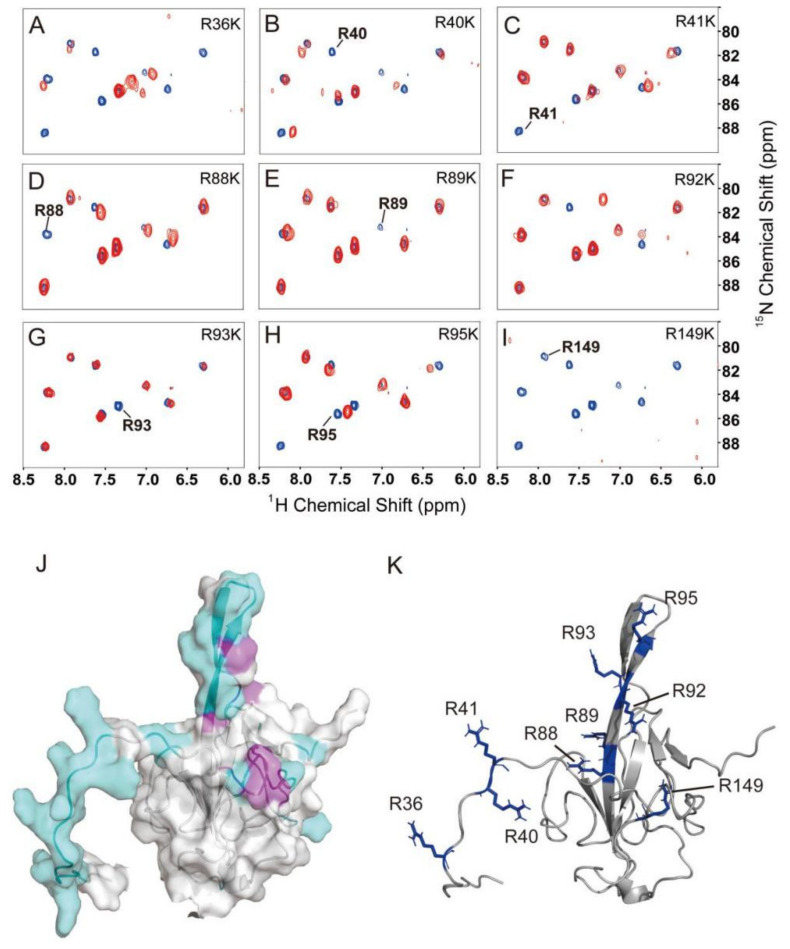
NMR analysis of Arg residues in the N-NTD/A48 complex. (**A**–**I**) Overlay of ^1^H-^15^N HSQC spectra of the WT N-NTD/A48 complex (blue) and mutant N-NTD/A48 complexes (red), showing the regions corresponding to Arg side chains. The mutants introduced in N-NTD are indicated in the upper right area of each spectrum. (**J**) Chemical shift variation (CSV) distribution mapped onto the structure of the N-NTD/A48 complex. The protein surface is colored by electrostatic potential (magenta: negative; cyan: positive). (**K**) Schematic diagram showing the positions of the 15 arginine residues mutated in N-NTD.

Three Arg residues (R36, R40, and R41) were located within the N-terminal IDR, and five additional Arg residues (R88, R89, R92, R93, and R95) were located within or near the basic finger region (Figure 3K). These newly detected Arg residue signals were expected to originate from these regions, suggesting their involvement in the interaction network of the complex.

To assign these Arg residues precisely, we designed fifteen single mutants in N-NTD, replacing arginine with lysine (R-K mutants). Because both residues bear positive charges, this conserved substitution generally does not substantially alter the surface charge distribution. We compared the spectra of each R-K mutant N-NTD complexed with A48 with that of the wild-type (WT) N-NTD-A48 complex (Supplementary Figure S5).

This approach yielded two potential outcomes. First, the mutant could cause the disappearance of a specific Arg side-chain cross-peak, allowing precise identification of Arg residues at the N-NTD-A48 interaction interface. Such residues might be important for interaction but not necessarily for determining the overall structure of the complex. Second, if a single mutant induced noticeable changes in the overall spectral pattern, this would indicate a more complex role for the affected Arg residue(s) and their surrounding region in determining the structural arrangement of the complex, underscoring their potential structural significance.

The spectra of the R88K, R89K, and R93K-N-NTD complexes with A48 lacked specific Arg side-chain signals compared with the WT N-NTD-A48 complex (Figure 3D,E,G). This absence allowed the assignment of these three Arg residues and indicated their involvement in A48 binding. These Arg residues are located within or near the basic finger region, consistent with the HSQC data of backbone amide signals, supporting the role of the basic finger in A48 interaction (Figure 3K). The R-K mutants did not induce significant changes in the remaining Arg signals, indicating that the overall structure of the complex was preserved. These observations confirm that these three Arg residues participate in A48 binding but are not critical determinants of the complex architecture.

The R36K, R40K, R41K, and R95K mutants induced substantial spectral changes in the N-NTD-A48 complex compared with the WT N-NTD-A48 complex (Figure 3A–C,H). In the R36K-N-NTD-A48 complex, most Arg side-chain signals, except that of R93, shifted or disappeared. In parallel, clear deviations in spectral patterns were evident in the backbone amide regions between the R36K-N-NTD-A48 and WT N-NTD-A48 complexes. These observations support a role for R36, located within the N-terminal IDR of N-NTD, in maintaining the overall structure of the complex. In the R40K, R41K, and R95K-N-NTD-A48 complexes, specific signals also disappeared. In the R40K complex, both R40 and R41 signals vanished, whereas the introduction of either the R41K or R95K mutant disrupted both R41 and R95 residues. This observation suggested a cooperative binding mode involving these residues in the interaction with A48. R36, R40, and R41 are all located within the N-terminal IDR of N-NTD. Thus, these findings indicate that Arg residues in the N-terminal IDR are hotspots that play important roles in both aptamer binding and determining the structural configuration of the complex. The perturbations caused by the conserved R-K mutants underscore the sensitivity of the complex structure to changes within the N-terminal IDR. This observation was consistent with conclusions drawn from the Δ43N-NTD-A48 complex analysis, reinforcing that minor alterations in the N-terminal IDR can exert substantial effects on the architecture of the complex.

Both the R92K and R149K mutants induced significant changes in the spectral patterns. The R92K mutant resulted in chemical shift changes in five arginine side chains (Figure 3F). However, the number of arginine side-chain signals remained unchanged from that of the WT N-NTD-A48 complex, suggesting that R92 is not directly involved in A48 binding. Instead, the mutant likely altered the local structure of the N-NTD-A48 complex, affecting residues near R92.

The R149K-N-NTD complex behaved differently. In the unbound state of WT N-NTD, a single signal was detected in the Arg side-chain region, whereas this signal was absent in R149K-N-NTD, allowing assignment of the R149 side chain in unbound N-NTD (Figure 3I). The corresponding cross-peak of R149 did not shift upon A48 addition to WT N-NTD, suggesting that R149 does not directly interact with A48. However, in the R149K-N-NTD-A48 complex, no characteristic Arg side-chain signals were observed in the HSQC spectra. This finding indicated that R149 plays a role in stabilizing both the N-NTD structure and the N-NTD-A48 complex, rather than acting as a residue that directly contributes to binding between N-NTD and A48.

In summary, analysis of the mutagenesis data provided insights into the distinct roles of individual Arg residues in the N-NTD-A48 complex. Most Arg residues in the basic finger region of N-NTD directly interact with A48 but do not strongly influence the overall structure of the complex. In contrast, Arg residues in the N-terminal IDR serve dual functions: they participate in direct interactions with A48 and also influence the overall topological arrangement of the N-NTD-A48 complex. This observation highlights the importance of the N-terminal IDR in aptamer recognition.

### 2.4. Surface Plasmon Resonance Assay Confirms the Putative DNA Binding Sites

**Figure 4:**
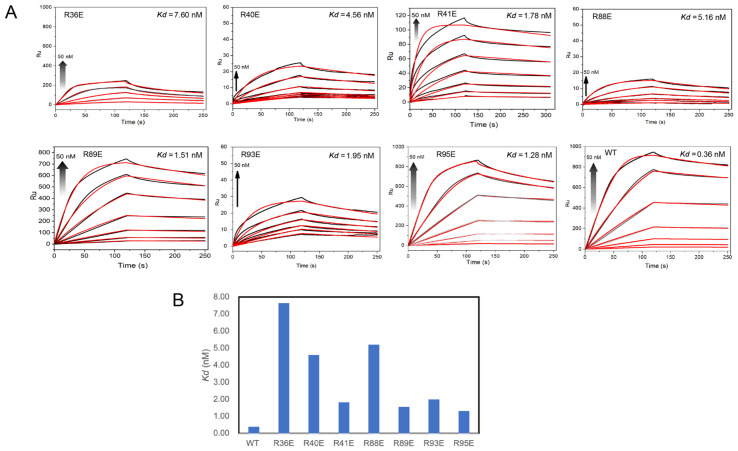
Surface plasmon resonance (SPR) analysis of binding between A48 and arginine-to-glutamate (R-E) mutants of N-NTD. (**A**) Sensorgrams of A48 binding to immobilized wild-type (WT) and R-E mutant N-NTD proteins. The black curves represent the raw experimental data, while the red lines indicate the globally fitted data derived from the kinetic model. All N-NTD proteins were used at a concentration of 50 μg/mL. (**B**) *Kd* of A48 binding to N-NTD and R-E mutants determined by SPR. Data are shown as response units (RUs) versus time. Experimental conditions: sodium acetate buffer, pH 4.5. Each sensorgram was recorded with a 3 min injection of N-NTD (R-E)/A48, followed by a 2 min association phase and a 2 min dissociation phase.

Based on the NMR identification of Arg residues involved in A48 binding, we generated seven arginine-to-glutamate (R-E) mutants and measured their binding affinities by SPR (Figure 4). Compared with WT N-NTD, all seven R-E mutants showed reduced binding to A48. The R36E mutant in the N-terminal IDR reduced binding affinity by 20-fold, and the R40E mutant reduced it by 12-fold. These results are consistent with the NMR data and indicate that Arg residues in the N-terminal IDR serve as hotspots for binding. Among the other R-E mutants, R88E showed a 14-fold decrease in binding affinity. This finding demonstrates that altering the charge of Arg residues affects the interaction between N-NTD and A48. Together, these SPR results support the NMR conclusion that Arg residues in the N-terminal IDR play an important role in mediating binding and influencing the affinity of the complex.

### 2.5. Structure of the N-NTD-A48 Complex

**Figure 5:**
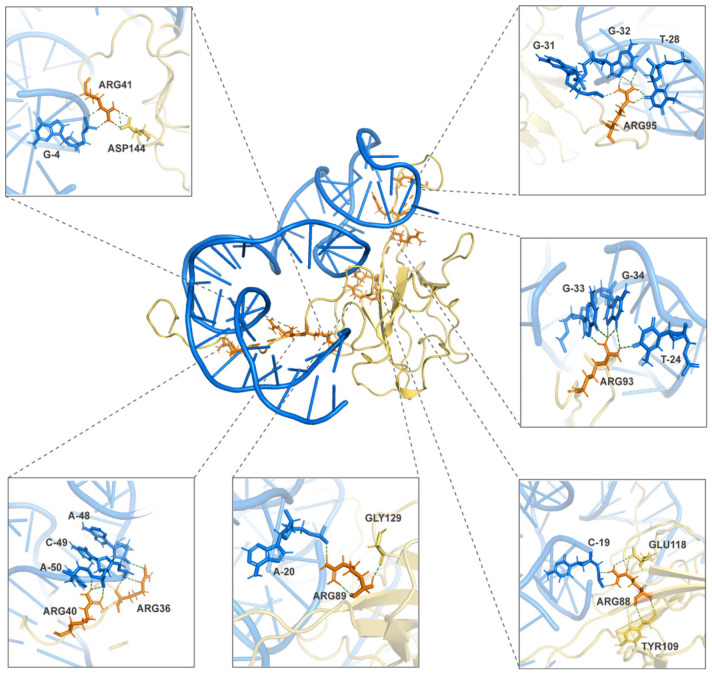
Hydrogen bonding interactions at key residue sites in the Δ25N-NTD-A48 complex. The Δ25N-NTD structure is shown in yellow, and A48 is shown in blue. Key arginine (Arg) residues are shown as orange sticks. Nucleotides that form hydrogen bonds with key Arg residues are shown as blue sticks, and amino acid residues that form hydrogen bonds with key Arg residues are shown as yellow sticks.

Based on the NMR data, we determined the structural model of the N-NTD-A48 complex using HADDOCK, followed by molecular dynamics refinement with AMBER (Figure 5). The N-terminus and the basic finger region are involved in interacting with A48. Within A48, residues 7–52 fold into an L-shaped overall topology. A large surface contact area was detected, involving the flexible N-terminal IDR, the basic finger, and part of the β-sheet.

Structural analysis of the complex reveals that the N-terminal residues R36 and R40 interact with A48 residues 48–50 through a combination of hydrophobic and hydrophilic contacts. In addition, residue R41 forms a hydrogen bond with residue G4 of A48. In addition to the N-terminus, A48 also interacts with other residues of N-NTD. Residue R88 forms a hydrogen bond with C9 of A48 and engages in multiple hydrogen bonds with neighboring residues, stabilizing the contact between the central β-sheet of N-NTD and the aptamer. Furthermore, the basic finger region of N-NTD interfaces with A48 through its basic residues and aptamer residues 24–34, resulting in multiple hydrophobic and hydrophilic interactions that stabilize the association between N-NTD and the aptamer. The Arg residues R36, R92, R107, and R149 of SARS-CoV-2 N-NTD are conserved among SARS-CoV-2 variants (Supplementary Figure S6B). This observation suggests that the aptamer may establish similar interactions with N proteins from other SARS-CoV-2 variants.

**Figure 6:**
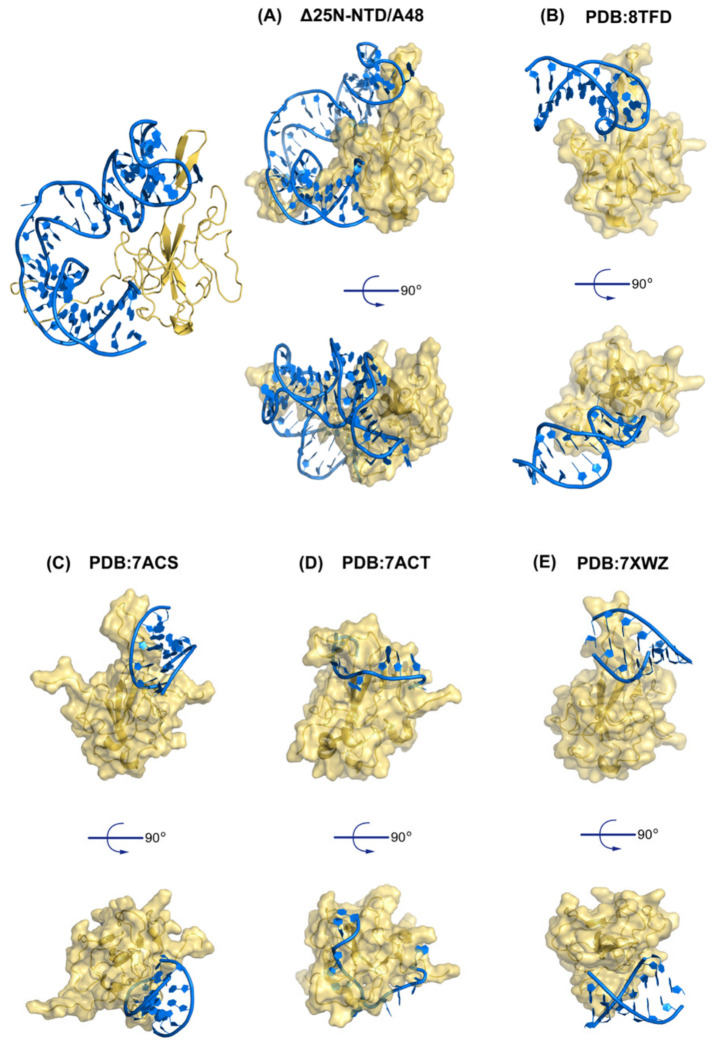
Comparison of the Δ25N-NTD-A48 complex with known N-NTD/RNA complex structures. The N-NTD structure is shown in yellow, and the nucleic acid (DNA or RNA) is shown in blue. (**A**) Binding model of the Δ25N-NTD-A48 complex. (**B**) binding model of the N-NTD(45–173)/A58-20 nt complex (PDB: 8 TFD). The A58-20 nt sequence is 5′-TCGGACATCGGATTGTCTGA-3′. (**C**) binding model of Δ43N-NTD with a 7 mer dsRNA (PDB: 7 ACS). The dsRNA sequences are 5′-CACUGAC-3′ and 5′-GUCAGUG-3′. (**D**) binding model of Δ43N-NTD with a 10 mer ssRNA (PDB: 7ACT). The ssRNA sequence is 5′-UCUCUAAACG-3′. (**E**) Binding model of Δ43N-NTD with a 7 mer dsRNA (PDB: 7 XWZ). The dsRNA sequences are 5′-CACUGAC-3′ and 5′-GUCAGUG-3′.

We further compared the structure of the Δ25N-NTD-A48 complex with the previously reported complex structures of N-NTD bound to A58–20 nt, single-stranded RNA (ssRNA), and double-stranded RNA (dsRNA) (2, 25, 26). These structural comparisons reveal that N-NTD adopts distinct binding modes when interacting with different ligands (Figure 6).

Comparison of the Δ25N-NTD-A48 complex with previously reported N-NTD–ligand structures revealed distinct binding patterns. In the Δ25N-NTD-A48 complex, A48 primarily binds to the left side of N-NTD, with the main binding interface involving the N-terminal region and the basic finger motif (Figure 6A). In the 8 TFD structure, the A58–20 nt truncation also binds predominantly to the left side of N-NTD near the basic finger motif; however, the top view reveals that a portion of its bases remain unbound and exposed. In the 7ACS complex, dsRNA mainly binds to the right side of N-NTD near the basic finger motif, occupying the front face of N-NTD with only limited contacts elsewhere, as seen from the top view. In the 7ACT complex, ssRNA predominantly encircles the basic finger motif, with its nucleobases oriented upward to engage the motif through intermolecular interactions. In the 7 XWZ complex, dsRNA also binds primarily to the basic finger motif, adopting an orientation relatively parallel to the front face of N-NTD.

Notably, these known N-NTD/aptamer complex structures were determined using N-terminally truncated NTD constructs and the role of the flexible N-terminal region in N-NTD/aptamer binding has not been addressed in earlier studies. The differences between the N-NTD/DNA and N-NTD/RNA complex structures indicate distinct interaction modes of N-NTD with these two types of nucleic acids. Therefore, understanding the binding modes of N-NTD with DNA is important for elucidating the overall nucleic acid binding mechanism of N-NTD.

## 3. Discussion

In this study, We determined the solution structure of the SARS-CoV-2 nucleocapsid protein N-terminal domain (N-NTD) bound to the DNA aptamer A48 by NMR spectroscopy. The structure reveals that both the intrinsically disordered region (IDR) and the basic finger of N-NTD participate in aptamer binding. Positively charged residues within the N-terminal IDR form multiple contacts with A48, and the IDR adopts a more ordered conformation upon binding, suggesting a coupled binding-and-folding mechanism.

Previous structural studies of coronavirus N proteins have typically used constructs lacking the flexible N-terminal IDR, leaving the functional role of this region in nucleic acid recognition unclear. Our findings demonstrate that the N-terminal IDR is not merely a passive linker but actively contributes to DNA binding. This observation may have general implications for understanding how other viral nucleocapsid proteins interact with nucleic acids.

Mutagenesis experiments combining SPR and NMR further support the importance of hydrophilic interactions in the N-NTD–A48 complex. Substitution of arginine with glutamate in the N-terminal IDR substantially reduced binding affinity, indicating that aptamer recognition is sensitive to sequence variations in this region. Given the high mutant rate of SARS-CoV-2, this sensitivity may influence aptamer binding across different viral variants.

Despite the genetic diversity of SARS-CoV-2 variants, the N protein sequence is highly conserved (Supplementary Figure S6). This conservation, together with the structural insights presented here, suggests that the N-terminal IDR and basic finger represent potential targets for aptamer-based diagnostics or therapeutics.

In summary, using liquid-state NMR spectroscopy, we identified amino acid residues at the interface between SARS-CoV-2 N-NTD and the A48 aptamer. Guided by NMR data and affinity analyses of single-point Arg mutants, our findings indicate that both the N-terminal IDR and the structured basic finger region of N-NTD are important for nucleic acid binding and recognition. Unlike many previous structural studies that excluded the flexible N-terminal segment, this work demonstrates that this region plays a key role in mediating nucleic acid binding. These findings suggest that the N-terminal IDR may represent a target for sequence-based drug design against viral infections.

## 4. Materials and Methods

### 4.1. Expression and Purification of SARS-CoV-2 Nucleocapsid Protein N-Terminal Domain and Aptamer

All DNA aptamers were obtained from the Beijing Genomics Institution (BGI). The DNA sequence of A48 is as follows: 5′-GCTGGATGTCGCTTACGACAATATTCCTTAGGGGC.

ACCGCTACATTGACACATCCAGC-3′. The isotopically labeled chemicals, including ^15^NH_4_Cl and ^13^C_6_-glucose, were purchased from Cambridge Isotope Laboratories (Andover, MA, USA).

The N-terminal domain of the SARS-CoV-2 nucleocapsid protein (N-NTD, residues 1–180), N-terminally truncated constructs Δ25N-NTD and Δ43N-NTD, and N-NTD mutants were expressed in *E. coli* BL21 (DE3). The genes encoding proteins with an N-terminal 6 × His tag were cloned into pET-28a. N-NTD mutagenesis was performed by overlap extension PCR.

The engineered strains were inoculated in Luria-Bertani broth (LB) containing 50 mg/mL Kanamycin and grown in a shaker at 220 rpm and 37 °C. Once the OD_600_ reached 0.6~0.8, the cells were collected by centrifugation at 9000× *g* for 5 min. The collected cells were suspended in an equal volume of M9 minimal medium. After incubating at 37 °C for 30 min, isopropylthio-β-galactoside (IPTG) was added to a final concentration of 0.5 mM to induce protein expression. The cells were harvested by centrifugation (9000× *g*, 5 min, 4 °C) 8 h after induction. To produce ^15^N,^13^C-labeled N-NTD, ^15^NH_4_Cl (1 g/L) and ^13^C_6_-glucose (4 g/L) were supplemented in M9 minimal medium as the sole nitrogen and carbon sources, respectively.

The harvested cell pellets were re-suspended in Buffer A (20 mM Tris, 2 M NaCl, 10% Glycerol, 1 mM PMSF, pH 7.4) supplemented with 1× protease inhibitor cocktail. The cells were disrupted by a high-pressure homogenizer at 4 °C, followed by centrifugation (4 °C, 10,000× *g*, 1 h) to remove the insoluble cell debris. The purification of N-NTD was carried out using Ni-NTA affinity chromatography (QIAGEN, Hilden, Germany) following a batch protocol. The supernatant was mixed with the Ni-NTA resin and incubated on ice for three hours. The beads were washed with buffer B (20 mM Tris, 2 M NaCl, 30 mM imidazole, pH 7.4) to remove impurities. The proteins were eluted using buffer C (20 mM Tris, 1 M NaCl, 300 mM imidazole, pH 7.4). The purified proteins were buffer-exchanged with NMR buffer (100 mM Na_2_HPO_4_, 50 mM NaCl, pH 7.4) through dialysis at 4 °C. The final yield of ^15^N,^13^C-N-NTD was approximately 50 mg per liter of cell culture.

### 4.2. Preparation of Deuterated N-NTD Sample 

The NMR assignments of the N-NTD-A48 complex were performed on a ^2^D,^15^N,^13^C-labeled N-NTD sample. To prepare the ^2^D-labeled N-NTD, the strain was gradually adapted in a D_2_O-based M9 minimal medium, starting from 50% D_2_O and incrementally increasing to 75%, 85%, 95%, and finally 99% D_2_O-based medium. The procedure for protein expression and purification of ^2^D,^15^N,^13^C-labeled N-NTD was the same as that used for the protonated sample. The final yield of ^2^D,^15^N,^13^C-labeled N-NTD was approximately 30 mg per liter of cell culture.

### 4.3. NMR Spectroscopy

All NMR experiments were performed on Bruker Avance3 spectrometers operating at 600 MHz, 700 MHz, and 900 MHz, each equipped with a cryo-H/C/N probe. The experiments were conducted at either 298 K or 308 K. The backbone assignments of N-NTD were accomplished using a series of triple resonance spectra, namely HNCA, HNCOCA, HNCACB, HNCOCACB, HNCO, and HNCACO. These spectra were acquired on a 0.5 mM sample of ^15^N,^13^C -labeled N-NTD at 308 K.

For the backbone assignments of the N-NTD-A48 complex, a 0.5 mM ^2^D,^15^N,^13^C -labeled N-NTD sample was mixed with A48 in a molar ratio of 1:1.2 (N-NTD vs. A48). To assign the sidechains of Arg residues, comparisons between the spectra of the mutated and WT N-NTD were performed. The secondary structure of N-NTD/A48 was determined using the CSI [27]. Additionally, CSV between N-NTD and the N-NTD-A48 complex was measured according to Equation, where ΔδH and ΔδN represented the chemical shift differences of the same residues between the N-NTD and N-NTD-A48 complex.

$$CSV=\sqrt{\frac{{\left(∆\delta H\right)}^{2}+{\frac{\left(∆\delta N\right)}{25}}^{2}}{2}}$$

The NMR titration experiments between N-NTD and A48 were conducted using a 0.2 mM sample of ^15^N-labeled N-NTD and unlabeled A48. The ^1^H-^15^N HSQC spectra of N-NTD were recorded both before and after the addition of A48, with the titration progressing up to a molar ratio of 1:1.2 (N-NTD:A48). All titration experiments were performed at either 308 K or 298 K. The acquired NMR spectra were processed using the Topspin 3.2 program and subsequently analyzed using the CARA program (version 1.9.1).

### 4.4. Haddock Calculation 

To perform the HADDOCK docking calculation, the secondary structure of the ssDNA of interest was predicted using mfold (version 3.6) [28]. Based on the related RNA tertiary structure obtained from MMB (version 1.0) [29], the RNA structure was converted to a DNA structure by modifying sugar residues and then relaxed using the AMBER program (version 18) [30].

The docking was carried out using the structural model of Δ25N-NTD, which was built from the crystal structure of truncated N-NTD (PDB code: 7CDZ, residue 44–180) with additions of the missing segments 26–43. The HADDOCK docking procedure was conducted using the HADDOCK web server (https://bianca.science.uu.nl/haddock2.4/ (accessed on 5 March 2023). The results of the docking were clustered, and the docked complex of N-NTD-A48 was identified based on docking scores. The N-NTD residues with CSP > 0.2 ppm and at least 15% relative solvent accessibility (RSA) were chosen as active residues (G30, G34, A35, R36, K38, Q39, R40, R41, P42, Q43, G44, L45, A50, S51, A55, A90, T91, R92, R93, I94, R95, G96, G97, D98, M101, K102, S105, N150, A152, N153, A155, Q163, G164, G170). Adjacent solvent-exposed residues were selected as passive residues (Q28, N29, E31, R32, S33, P46, N47, N48, T49, W52, H59, G60, K61, E62, P67, Q70, I74, R88, R89, G99, K100, D103, L104, P106, R107, Y109, P117, G129, G147, T148, N150, P151, L161, P162, T165, T166, L167, P168, K169, F171, Y172). All surface residues within 6.50 Å around the active residues were also selected. The N-terminal region of N-NTD (N-terminal residues G25 to L45) was defined as a fully flexible segment during the docking. The active residues of the experiment-driven approach in the A48 aptamer were defined as residues 7–52. A fraction (2%) of the ambiguous restraints (AIRs) was randomly excluded to add some flexibility to the docking process. The clustering method used was the Fraction of Common Contacts (FCC) with a default cut-off of 0.60 and a minimal cluster size of 10. After the docking simulations, the structures were analyzed using PyMOL 3.1 [31]. The structure with the lowest interaction energy was selected for further detailed analysis and visualization.

### 4.5. Molecular Dynamic Simulation

The N-NTD-A48 complex system was neutral and solvated in a periodic TIP3P water box. All molecular dynamics simulation runs were performed using AMBER 19 package [32] with ff14SB [33] protein force field and OL15 DNA force field [34]. The van der Waals cutoff was set to 10 Å, and explicit solvent particle mesh Ewald was used for long-range electrostatic energy calculation. Minimization, heating, and holding processes were performed before production runs. All production simulations were performed at 300 K under the NPT ensemble with an integration step of 2 fs and anisotropic pressure coupling. Seven independent runs for the system. Production trajectories of 600 ns were generated for all simulations. Water molecules of all trajectories were removed first before analysis. Hydrogen bond analyses were performed using Gnuplot (version 5.4).

### 4.6. Surface Plasmon Resonance Experiments

SPR experiments were conducted using a BIAcore T200 biosensor system (GE Healthcare, Marlborough, MA, USA) to determine the binding affinity of R to E mutants of N-NTD and the A48 aptamer. To immobilize the protein on the sensor chip, a carboxymethylated sensor chip (CM5 chip) was used. The carboxylic groups on the chip were activated using the standard amine coupling procedure with freshly prepared EDC/NHS. Then, the R-E mutants (50 µg/mL) in acetate buffer (pH 4.5) were injected onto the sensor chip at a flow rate of 5 µL/min until reaching approximately 500 Response Units (RU) immobilization level. To block any unreacted carboxyl groups, deactivation was performed using ethanolamine-HCl. The binding analysis was carried out with the A48 aptamer at various concentrations, ranging from 5 to 500 nM. The running conditions were set at a flow rate of 30 µL/min, at 25 °C, with 2 min of association time and 2 min of dissociation time. The running buffer used was DPBS (137 mM NaCl, 8 mM Na_2_HPO_4_, 2.7 mM KCl, 1.5 mM KH_2_PO_4_). For regeneration of the sensor chip, a flow rate of 30 µL/min, 25 °C, and 30 s was used with a regeneration buffer containing 1.5 M NaCl and 10 mM NaOH. All buffers were filtered and degassed before the experiments. The sample injection mode employed was multi-cycle kinetic injection. Upon injection of the A48 aptamer, sensorgrams recording the association/dissociation behavior of the R-E mutants/A48 complex were collected. To obtain the final binding dissociation curve, all data were corrected with the binding curve obtained without adding the aptamer.

To calculate the *Kd*, a series of sensorgrams were collected at various aptamer concentrations and subsequently analyzed using the 1:1 Langmuir binding model within the BIA evaluation software (version 3.1).

## Data Availability

The original contributions presented in this study are included in the article/Supplementary Material. Further inquiries can be directed to the corresponding author(s).

## Supplementary Materials

The following supporting information can be downloaded at: https://www.mdpi.com/article/10.3390/ijms27146386/s1.

## References

[1] Lu S., Ye Q., Singh D., Cao Y., Diedrich J.K., Yates J.R., Villa E., Cleveland D.W., Corbett K.D. (2021). The SARS-CoV-2 nucleocapsid phosphoprotein forms mutually exclusive condensates with RNA and the membrane-associated M protein. Nat. Commun..

[2] Dinesh D.C., Chalupska D., Silhan J., Koutna E., Nencka R., Veverka V., Boura E. (2020). Structural basis of RNA recognition by the SARS-CoV-2 nucleocapsid phosphoprotein. PLoS Pathog..

[3] Cubuk J., Alston J.J., Incicco J.J., Singh S., Stuchell-Brereton M.D., Ward M.D., Zimmerman M.I., Vithani N., Griffith D., Wagoner J.A. (2021). The SARS-CoV-2 nucleocapsid protein is dynamic, disordered, and phase separates with RNA. Nat. Commun..

[4] Forsythe H.M., Rodriguez Galvan J., Yu Z., Pinckney S., Reardon P., Cooley R.B., Zhu P., Rolland A.D., Prell J.S., Barbar E. (2021). Multivalent binding of the partially disordered SARS-CoV-2 nucleocapsid phosphoprotein dimer to RNA. Biophys. J..

[5] Hadjadj J., Yatim N., Barnabei L., Corneau A., Boussier J., Smith N., Pere H., Charbit B., Bondet V., Chenevier-Gobeaux C. (2020). Impaired type I interferon activity and inflammatory responses in severe COVID-19 patients. Science.

[6] Lei X., Dong X., Ma R., Wang W., Xiao X., Tian Z., Wang C., Wang Y., Li L., Ren L. (2020). Activation and evasion of type I interferon responses by SARS-CoV-2. Nat. Commun..

[7] Zhao Y., Sui L., Wu P., Wang W., Wang Z., Yu Y., Hou Z., Tan G., Liu Q., Wang G. (2021). A dual-role of SARS-CoV-2 nucleocapsid protein in regulating innate immune response. Signal Transduct. Target. Ther..

[8] Gao T., Hu M., Zhang X., Li H., Zhu L., Liu H., Dong Q., Zhang Z., Wang Z., Hu Y. (2020). Highly pathogenic coronavirus N protein aggravates lung injury by MASP-2mediated complement over-activation. medRxiv.

[9] Mangalmurti N., Hunter C.A. (2020). Cytokine Storms: Understanding COVID-19. Immunity.

[10] Mu J., Fang Y., Yang Q., Shu T., Wang A., Huang M., Jin L., Deng F., Qiu Y., Zhou X. (2020). SARS-CoV-2 N protein antagonizes type I interferon signaling by suppressing phosphorylation and nuclear translocation of STAT1 and STAT2. Cell Discov..

[11] Wu S.-W., Chen Y.-J., Chang Y.-W., Huang C.-Y., Liu B.-H., Yu F.-Y. (2024). Novel enzyme-linked aptamer-antibody sandwich assay and hybrid lateral flow strip for SARS-CoV-2 detection. J. Nanobiotechnol..

[12] Çam Derin D., Gündüz E. (2025). Multiple detection method for SARS-CoV-2 with aptamer cocktails depending on their localization in paper-based assays. BMC Infect. Dis..

[13] Zhou S., Xu Y., Liao H., Ou H., Qi D., Wu Y., Liu Y., Li J., Li J., Shi B. (2026). Dual-mode aptamer-driven biosensing platform for ultrasensitive and mutation-resilient detection of the SARS-CoV-2 nucleocapsid protein. Genes Dis..

[14] Huang Y., Huang C., Chen J., Chen S., Li B., Li J., Jin Z., Zhang Q., Pan P., Du W. (2024). Inhibition of SARS-CoV-2 replication by a ssDNA aptamer targeting the nucleocapsid protein. Microbiol. Spectr..

[15] Zhang X., Guan L., Wang H., Liu J., Wei X., Xu Z., Wang S., Qin Q. (2025). Generation and application of novel DNA aptamers targeted to the nucleocapsid protein of the SARS-CoV-2. Virology.

[16] Zhang L., Fang X., Liu X., Ou H., Zhang H., Wang J., Li Q., Cheng H., Zhang W., Luo Z. (2020). Discovery of sandwich type COVID-19 nucleocapsid protein DNA aptamers. Chem. Commun..

[17] Chen Z., Wu Q., Chen J., Ni X., Dai J. (2020). A DNA Aptamer Based Method for Detection of SARS-CoV-2 Nucleocapsid Protein. Virol. Sin..

[18] Zhang X.H., Zhang X., Xu A.Q., Yu M.D., Xu Y., Xu Y., Wang C., Yang G.G., Song C.X., Wu X.W. (2022). Aptamer-Gated Mesoporous Silica Nanoparticles for N Protein Triggered Release of Remdesivir and Treatment of Novel Coronavirus (2019-nCoV). Biosensors.

[19] Li H.X., Fu X.Y., You Q.M., Shi D.W., Su L.X., Song M.H., Peng R.Z., Fu T., Wang P., Tan W.H. (2025). Multiple aptamer recognition-based quantum dot lateral flow platform: Ultrasensitive point-of-care testing of respiratory infectious diseases. J. Mater. Chem. B.

[20] Peng Y., Du N., Lei Y., Dorje S., Qi J., Luo T., Gao G.F., Song H. (2020). Structures of the SARS-CoV-2 nucleocapsid and their perspectives for drug design. EMBO J..

[21] Bouhaddou M., Memon D., Meyer B., White K.M., Rezelj V.V., Correa Marrero M., Polacco B.J., Melnyk J.E., Ulferts S., Kaake R.M. (2020). The Global Phosphorylation Landscape of SARS-CoV-2 Infection. Cell.

[22] Wu C.H., Chen P.J., Yeh S.H. (2014). Nucleocapsid phosphorylation and RNA helicase DDX1 recruitment enables coronavirus transition from discontinuous to continuous transcription. Cell Host Microbe.

[23] Davidson A.D., Williamson M.K., Lewis S., Shoemark D., Carroll M.W., Heesom K.J., Zambon M., Ellis J., Lewis P.A., Hiscox J.A. (2020). Characterisation of the transcriptome and proteome of SARS-CoV-2 reveals a cell passage induced in-frame deletion of the furin-like cleavage site from the spike glycoprotein. Genome Med..

[24] Chen X., He S., Xue S., Luo Y., Lu Z., Zhu S., Miao Z., Chen S., Huang L. (2026). Structural basis of DNA aptamer A58 targeting the N-terminal domain of sarbecoviruses nucleocapsid protein. J. Biol. Chem..

[25] Luan X.D., Li X.M., Li Y.F., Su G.C., Yin W.C., Jiang Y., Xu N., Wang F., Cheng W., Jin Y. (2022). Antiviral drug design based on structural insights into the N-terminal domain and C-terminal domain of the SARS-CoV-2 nucleocapsid protein. Sci. Bull..

[26] Esler M.A., Belica C.A., Rollie J.A., Brown W.L., Moghadasi S.A., Shi K., Harki D.A., Harris R.S., Aihara H. (2024). A compact stem-loop DNA aptamer targets a uracil-binding pocket in the SARS-CoV-2 nucleocapsid RNA-binding domain. Nucleic Acids Res..

[27] Wishart D.S., Sykes B.D., Richards F.M. (1992). The chemical shift index: A fast and simple method for the assignment of protein secondary structure through NMR spectroscopy. Biochemistry.

[28] http://unafold.rna.albany.edu.

[29] Flores S.C., Sherman M.A., Bruns C.M., Eastman P., Altman R.B. (2011). Fast Flexible Modeling of RNA Structure Using Internal Coordinates. IEEE-Acm Trans. Comput. Biol. Bioinform..

[30] http://ambermd.org/index.php.

[31] https://pymol.org/2/.

[32] Case D.A., Aktulga H.M., Belfon K., Cerutti D.S., Cisneros G.A., Cruzeiro V.W.D., Forouzesh N., Giese T.J., Götz A.W., Gohlke H. (2023). AmberTools. J. Chem. Inf. Model..

[33] Maier J.A., Martinez C., Kasavajhala K., Wickstrom L., Hauser K.E., Simmerling C. (2015). ff14SB: Improving the Accuracy of Protein Side Chain and Backbone Parameters from ff99SB. J. Chem. Theory Comput..

[34] Galindo-Murillo R., Robertson J.C., Zgarbová M., Šponer J., Otyepka M., Jurečka P., Cheatham T.E. (2016). Assessing the Current State of Amber Force Field Modifications for DNA. J. Chem. Theory Comput..

