# Supplementary material for: The Intrinsic Disordered N-Terminus of Nucleocapsid Protein of SARS-CoV-2 Is Critical in DNA Aptamer Binding
Source: Int J Mol Sci. 2026 Jul 18;27(14):6386. doi: 10.3390/ijms27146386 (PMC13409943; doi:10.3390/ijms27146386)
Supplement: Supplementary file 1 [file ijms-27-06386-s001.zip › ijms-4386241-supplementary.pdf]

Supporting Information

The intrinsic disordered N-terminus of nucleocapsid protein of SARS-CoV-2 is critical in DNA aptamer binding

Hongye Lu[1], Jiawen Ma[1], Xiaomin Ma[2], Yuanpeng Wu[1], Xuan Sun[3], Changxing Ma[1], Xiaoxian Li[1], Zhiyong Xu[1], Pengxi Lu[1], Zhaofeng Luo[4], Lixin Zhang[1,*], Liyun Zhang[5], Shenlin Wang[1,6,*]

Affiliations:

[1] State Key Laboratory of Bioreactor Engineering, East China University of Science and Technology, Shanghai 200237, China

[2] Instrumental Analysis and Research Center, Sun Yan-sen University, Guangzhou, Guangdong 510006, China

[3] State Key Laboratory of Microbial Metabolism, Joint International Research Laboratory of Metabolic and Developmental Sciences, School of Life Sciences and Biotechnology, Shanghai Jiao Tong University, Shanghai 200240, China

[4] School of Life Sciences, University of Science and Technology of China, Hefei, Anhui 230027, China

[5] State Key Laboratory of Medicinal Chemical Biology, Nankai University, Tianjin 300071, China

[6] Beijing NMR Center and College of Chemistry and Molecular Engineering, Peking University, Beijing 100871, China

*Corresponding authors: Prof. Lixin Zhang and Prof. Shenlin Wang

E-mails: wangshenlin@ecust.edu.cn

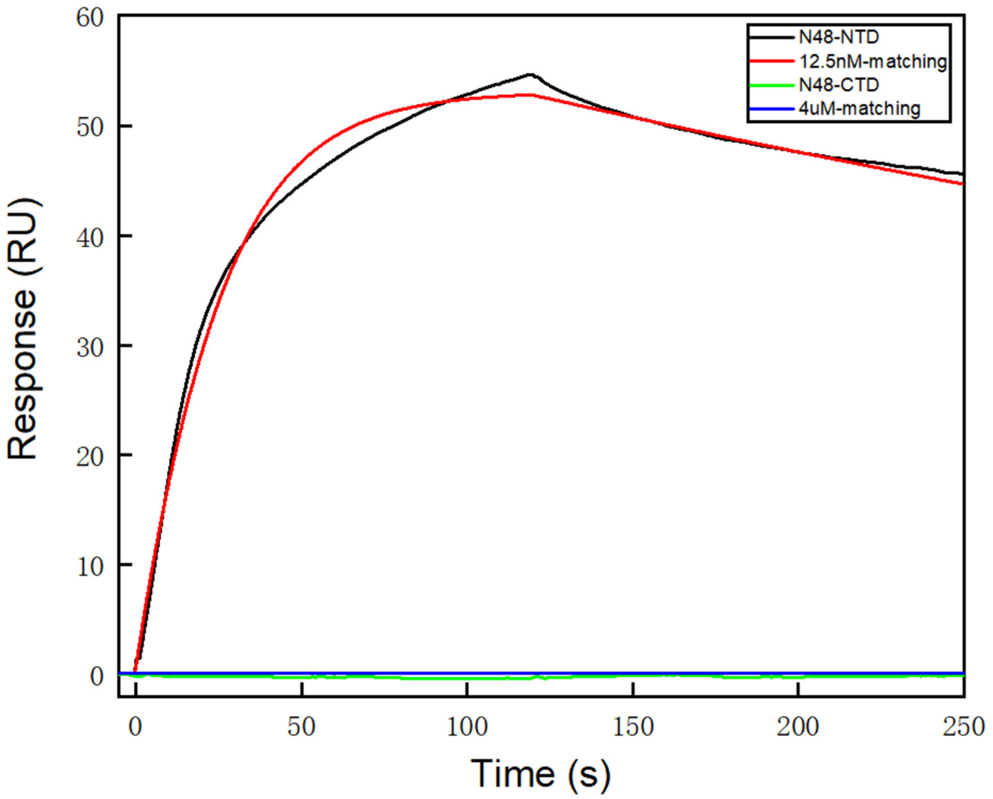

Supplementary Figure S1. SPR analysis of A48 binding to N-NTD and N-CTD.

Comparison of sensorgrams of A48 binding to N-NTD and N-CTD. Red lines indicate the fitted data for A48 binding to N-NTD, and blue lines indicate the fitted data for A48 binding to N-CTD. Data are shown as response units (RU) versus time. Experimental conditions: sodium acetate buffer (pH 4.5); N-NTD, 12.5 nM; N-CTD, 4 μM.

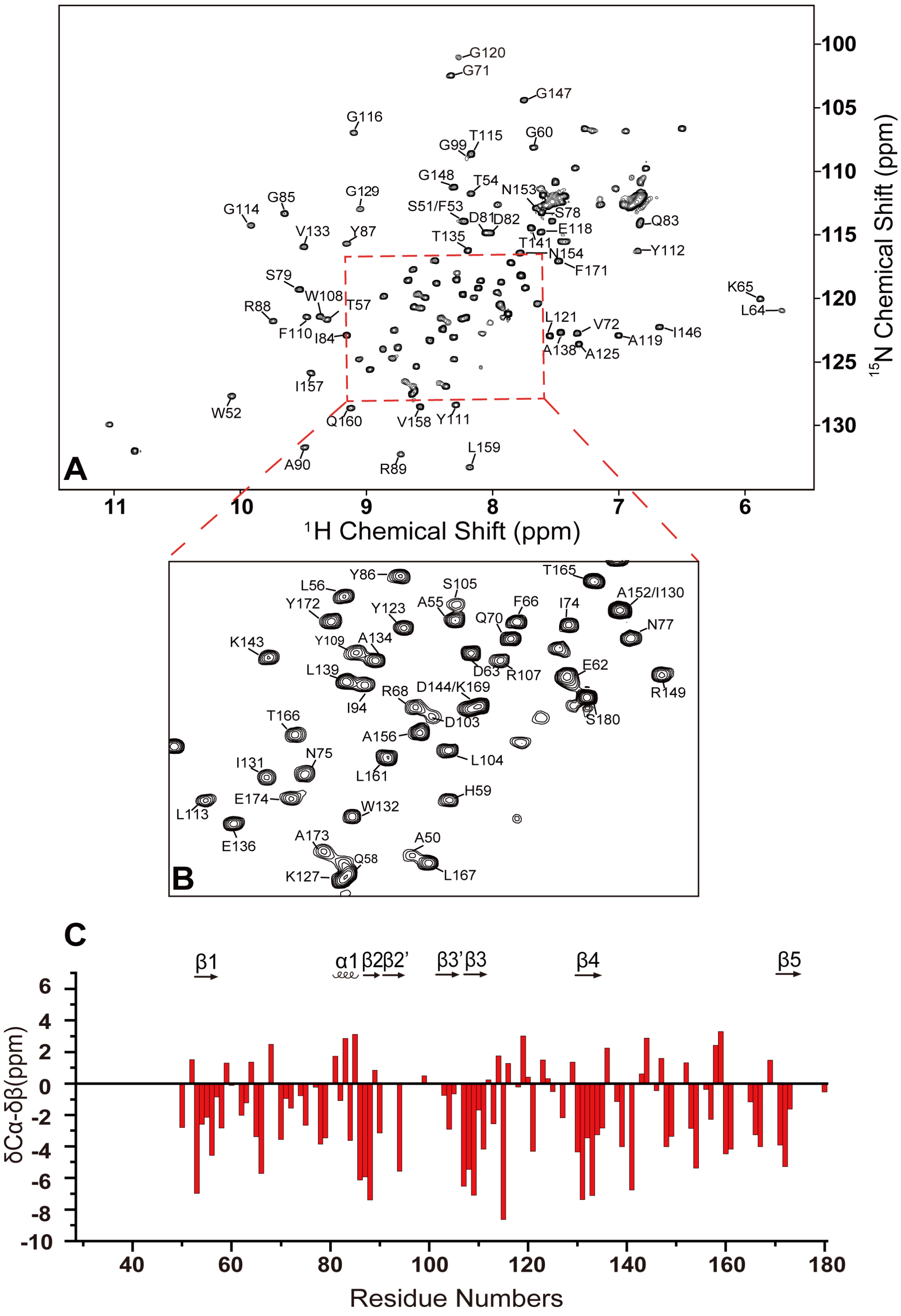

Supplementary Figure S2. ^{1}H-^{15}N HSQC spectrum and NMR assignments of N-NTD.

A, 2D ^{1}H-^{15}N HSQC spectrum of N-NTD acquired at a ^{1}H frequency of 700 MHz with assigned resonances indicated.

B, enlarged central region of the ^{1}H-^{15}N HSQC spectrum of N-NTD.

C, plot of CSI values versus residue number. The secondary structure derived from CSI values is shown above the plot.

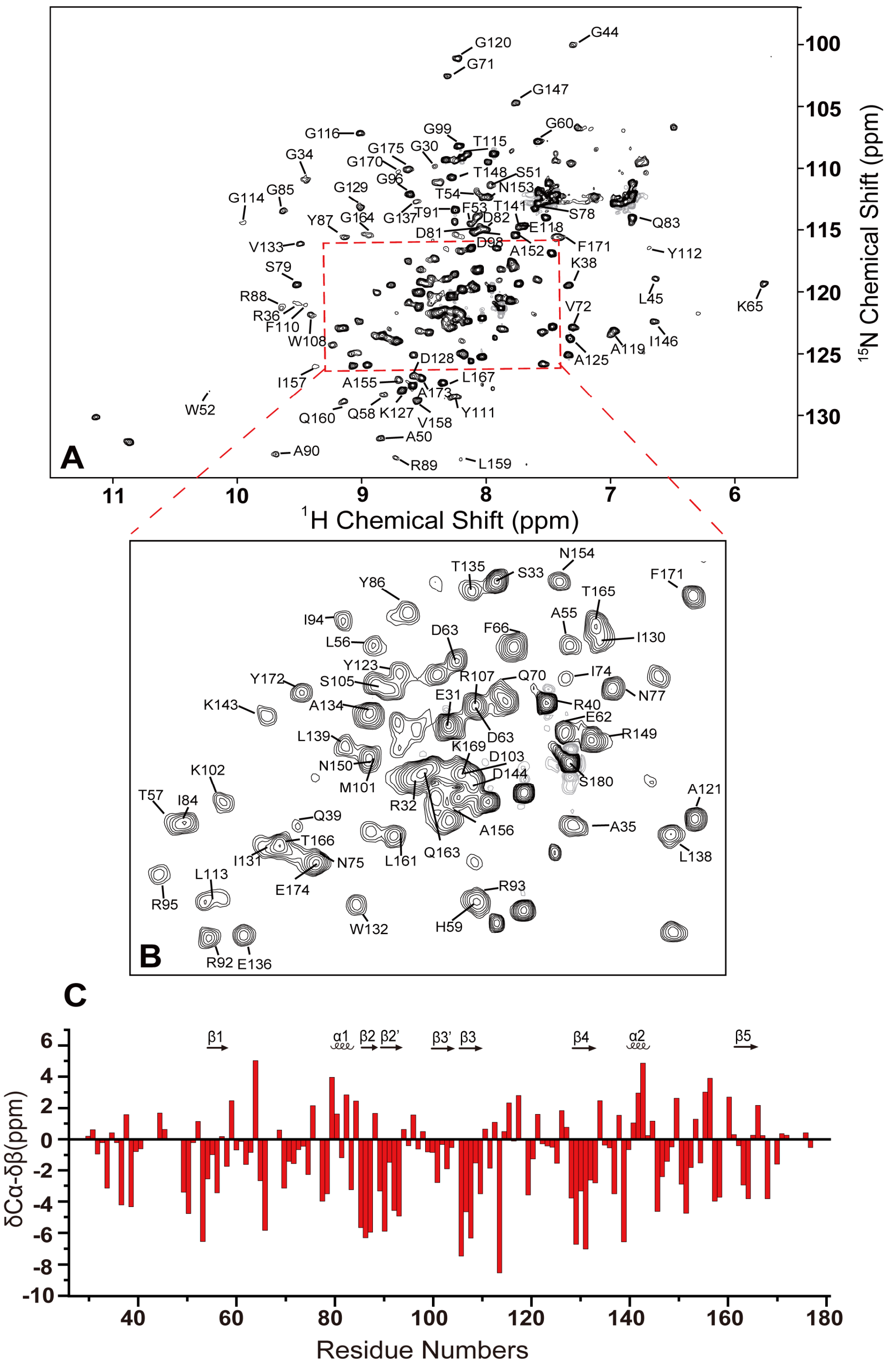

Supplementary Figure S3. ^{1}H-^{15}N HSQC spectrum and NMR assignments of the N-NTD-A48 complex.

A, 2D ^{1}H-^{15}N HSQC spectrum of the N-NTD-A48 complex acquired at a ^{1}H frequency of 700 MHz with assigned resonances indicated.

B, enlarged central region of the ^{1}H-^{15}N HSQC spectrum of the N-NTD-A48 complex.

C, plot of CSI values versus residue number. The secondary structure derived from CSI values is shown above the plot.

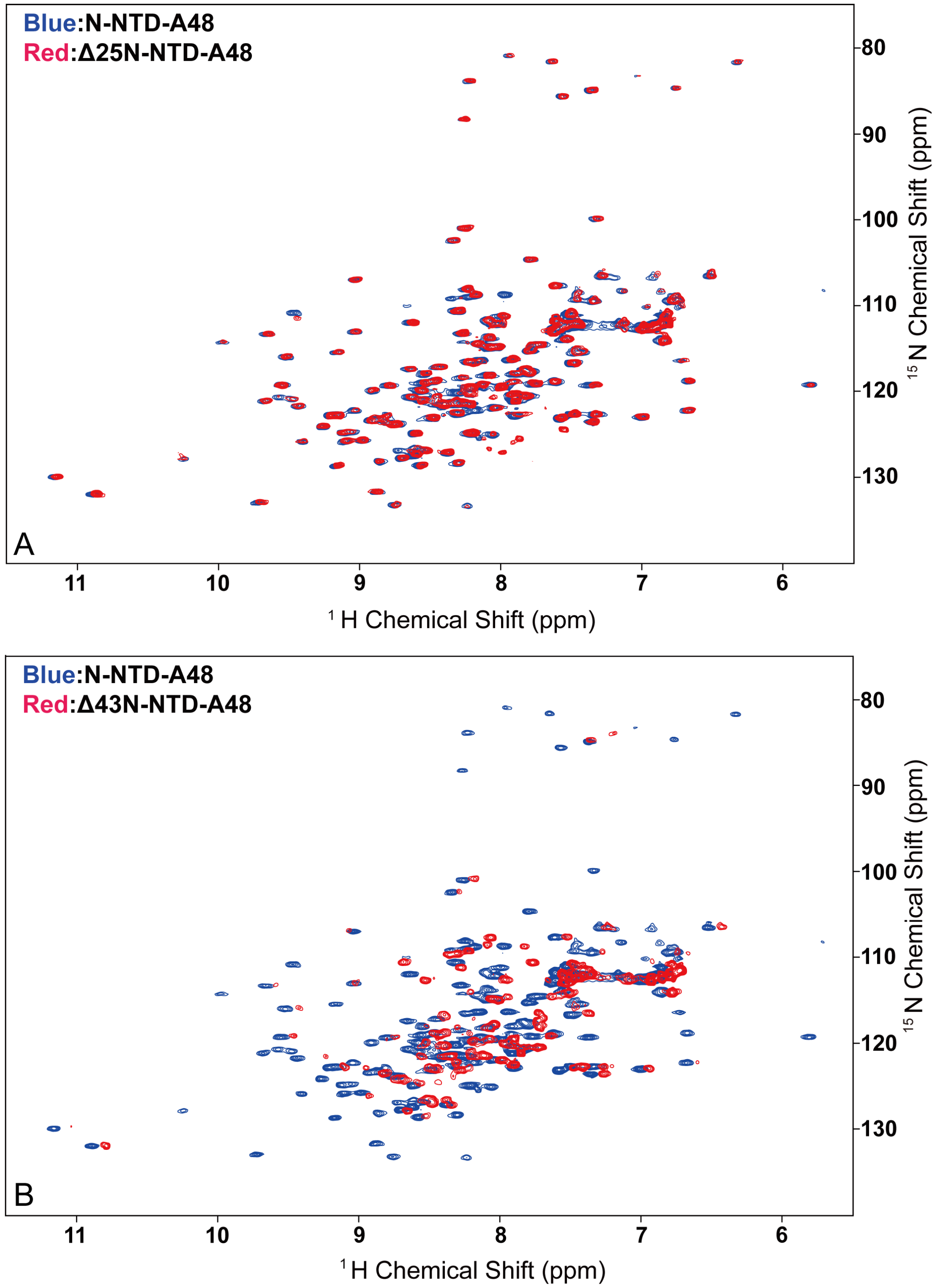

Supplementary Figure S4. Comparison of ^{1}H-^{15}N HSQC spectra of the N-NTD-A48 complex and N-terminus truncation forms of N-NTD in complex with A48.

A, overlay of HSQC spectra of WT N-NTD-A48 (blue) and Δ25-N-NTD-A48 (red).

B, overlay of HSQC spectra of WT N-NTD-A48 (blue) and Δ43-N-NTD-A48 (red).

Regions containing Arg side chains are shown.

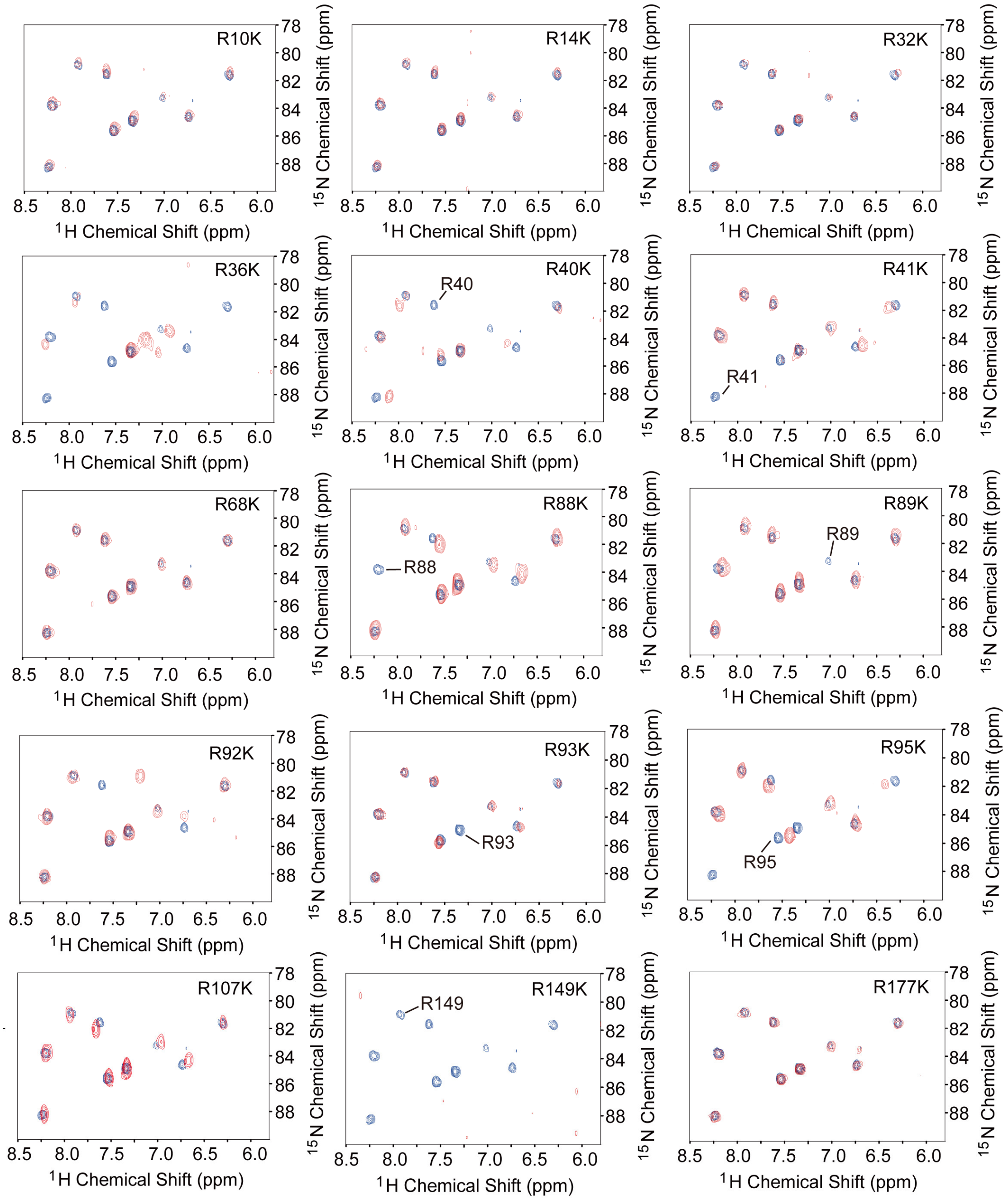

Supplementary Figure S5. Overlay of ^{1}H-^{15}N HSQC spectra of the N-NTD-A48 complex (blue) and the R-K mutated N-NTD-A48 complex (red), showing regions corresponding to Arg side chains.

The labels at the top-right of each subfigure indicate the positions of the mutants. NMR assignments of Arg side chains were made by comparison between the WT and N-NTD mutant, both bound to A48. Assignments are indicated in the subfigures.

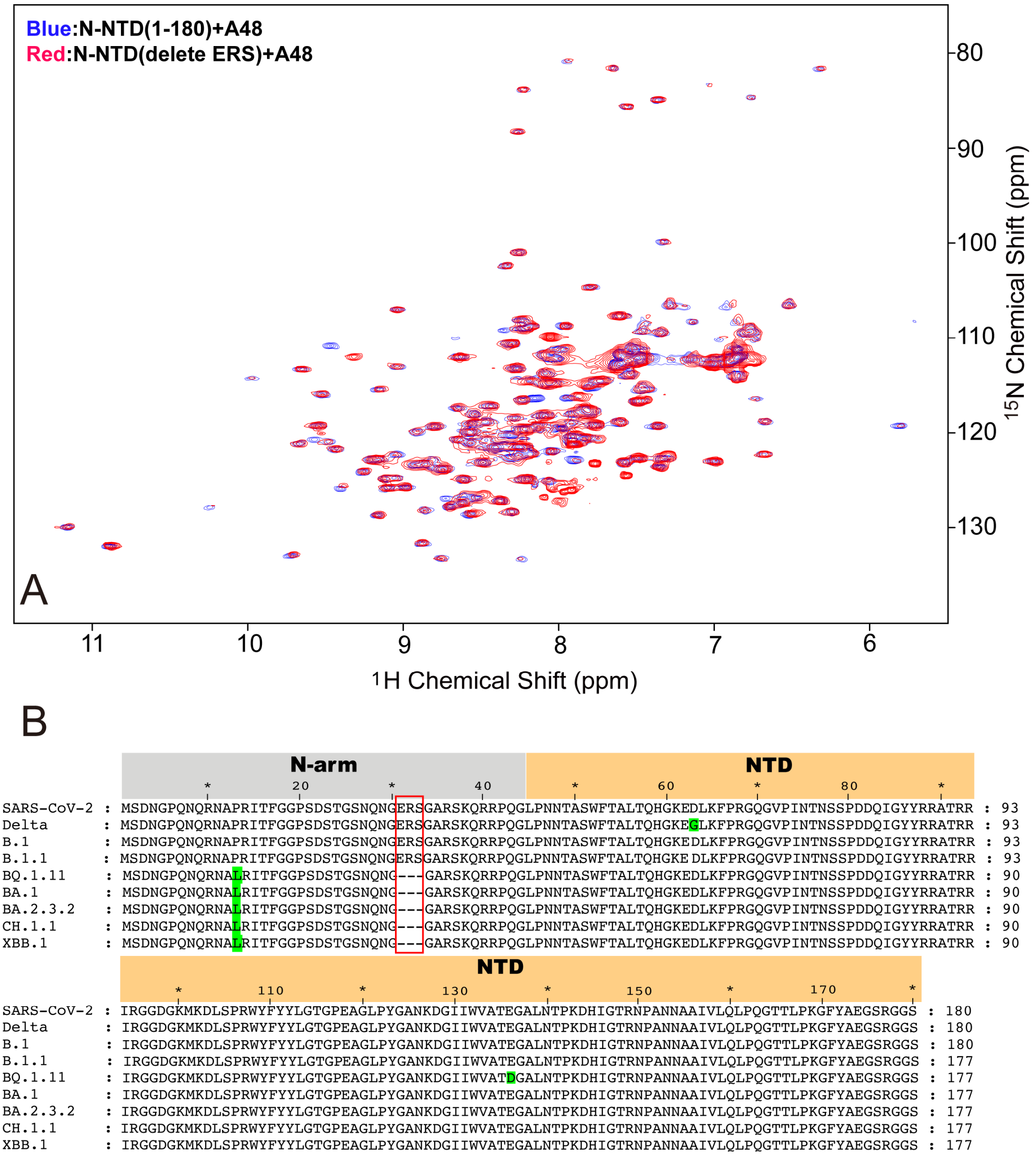

Supplementary Figure S6. Comparison of HSQC spectra of the ΔERS-N-NTD-A48 complex and the N-NTD-A48 complex and sequence alignment of SARS-CoV-2 N-NTD with its representative variants.

A, comparison of HSQC spectra of the ΔERS-N-NTD-A48 complex (red) and the N-NTD-A48 complex (blue).

B, sequence alignment of SARS-CoV-2 N-NTD with its representative variants. The alignment shows that the primary locations of variant mutations within the N-NTD region are P13, E31, R32, and S33 in the N-arm region. Green indicates residues that are not conserved with the SARS-CoV-2 N-NTD sequence
